# A multi-center study on the regenerative effects of erythropoietin in burn and scalding injuries: study protocol for a randomized controlled trial

**DOI:** 10.1186/1745-6215-14-124

**Published:** 2013-05-03

**Authors:** Christina Irene Günter, Augustinus Bader, Ulf Dornseifer, Silvia Egert, Sebastian Dunda, Gerrit Grieb, Thomas Wolter, Norbert Pallua, Tobias von Wild, Frank Siemers, Peter Mailänder, Oliver Thamm, Carsten Ernert, Michael Steen, Reiner Sievers, Bert Reichert, Afshin Rahmanian-Schwarz, Hans Schaller, Bernd Hartmann, Max Otte, Victoria Kehl, Christian Ohmann, Wolfgang Jelkmann, Hans-Günther Machens

**Affiliations:** 1 Coordinating Investigator: “EPO in Burns”, Clinic for Plastic Surgery and Hand Surgery, Klinikum Rechts der Isar, Technische Universität München, Ismaninger Str. 22, Munich, 81675, Germany; 2 Institute for Cell Techniques and Applied Stem Cell Biology, University of Leipzig, Deutscher Platz 5, Leipzig, 04103, Germany; 3 Klinik für Plastische, Rekonstruktive, Hand- und Verbrennungschirurgie, Klinikum Bogenhausen, Englschalkinger Str. 77, Munich, 81925, Germany; 4 Department of Plastic Surgery, Hand Surgery, Burn Center, University Hospital of the RWTH Aachen University, Pauwelsstr. 30, Aachen, 52074, Germany; 5 Sektion für Plastische Chirurgie und Handchirurgie, Zentrum für Schwerbrandverletzte, Universitätsklinikum Schleswig Holstein, Lübeck, 23538, Germany; 6 Abteilung für Plastische, Rekonstruktive und Handchirurgie, Krankenhaus Merheim, Ostmerheimer Str. 200, Köln, 51109, Germany; 7 Klinik für Plastische und Handchirurgie, Brandverletztenzentrum, BG-Kliniken Bergmannstrost Halle, Halle, 06112, Germany; 8 Klinik für Plastische, Wiederherstellende und Handchirurgie, Zentrum für Schwerbrandverletzte, Klinikum Nürnberg-Süd, Breslauer Strasse 201, Nürnberg, 90471, Germany; 9 Klinik für Hand-, Plastische, Rekonstruktive und Verbrennungschirurgie, Berufsgenossenschaftliche Unfallklinik, Tübingen, 72076, Germany; 10 Zentrum für Schwerbrandverletzte mit Plastischer Chirurgie, Unfallkrankenhaus Berlin, Berlin, 12683, Germany; 11 Klinik für Hand-, Plastische und Rekonstruktive Chirurgie, Schwerbrand Verletztenzentrum, Berufsgenossenschaftliche Unfallklinik, Ludwigshafen, 67071, Germany; 12 Institute of Medical Statistics and Epidemiology, Klinikum rechts der Isar, Technische Universität München, Ismaninger Str. 22, Munich, 81675, Germany; 13 Coordination Centre for Clinical Studies, Heinrich-Heine-University, Moorenstr. 5, Düsseldorf, 40225, Germany; 14 Institute for Physiology, University of Lübeck, Ratzeburger Allee 160, Lübeck, 23562, Germany; 15 Müncher Studienzentrum, Klinikum rechts der Isar, Technische Universität München, Ismaninger Str. 22, Munich, 81675, Germany

**Keywords:** Erythropoietin (EPO), Burns, Regenerative medicine, Wound healing, Clinical trial, Randomization

## Abstract

**Background:**

Although it was initially assumed that erythropoietin (EPO) was a hormone that only affected erythropoiesis, it has now been proposed that EPO plays an additional key role in the regulation of acute and chronic tissue damage.

Via the inhibition of inflammatory reactions and of apoptosis, stem cell recruitment, advancement of angiogenesis and growth factor release, EPO enhances healing and thus *restitutio ad integrum* after trauma. Human skin contains EPO receptors and is able to synthesize EPO. We therefore hypothesize that EPO is able to optimize wound healing in thermally injured patients.

**Methods/Design:**

This is a large, prospective, randomized, double-blind, multi-center study, funded by the German Federal Ministry of Education and Research, and fully approved by the designated ethics committee. The trial, which is to investigate the effects of EPO in severely burned patients, is in its recruitment phase and is being carried out in 13 German burn care centers. A total of 150 patients are to be enrolled to receive study medication every other day for 21 days (EPO 150 IU/kg body weight or placebo). A follow-up of one year is planned. The primary endpoint of this study is the time until complete re-epithelialization of a defined skin graft donor site is reached. Furthermore, clinical parameters such as wound healing, scar formation (using the Vancouver scar scale), laboratory values, quality of life (SF-36), angiogenic effects, and gene- and protein-expression patterns are to be determined. The results will be carefully evaluated for gender differences.

**Discussion:**

We are seeking new insights into the mechanisms of wound healing in thermally injured patients and more detailed information about the role EPO plays, specifically in these complex interactions. We additionally expect that the biomimetic effects of EPO will be useful in the treatment of acute thermal dermal injuries.

**Trial registration:**

EudraCT Number: 2006-002886-38, Protocol Number: 0506, ISRCT Number: http://controlled-trials.com/ISRCTN95777824/ISRCTN95777824.

## Background

Over the past few years, our understanding of erythropoietin (EPO) has changed significantly. Although it was initially assumed that EPO was a hormone affecting erythropoiesis only [1], it has now been discussed that EPO plays a key role in the repair of acute and chronic tissue damage. It has also been proposed that EPO has a significant anti-inflammatory effect, whereby the inflammatory response is part of the normal reaction of any organism to injury. Via the inhibition of inflammatory reactions, EPO influences healing and thus promotes *restitutio ad integrum* after trauma [2].

In addition, EPO seems to be involved in ensuring that stem cells are recruited to the required location. The best-known effect is the endothelial nitric oxide synthase (eNOS) mediated response and the recruitment of endothelial progenitor cells (EPCs) [3]. *In vitro* data show enhancement of stem cell proliferation and stem cell phenotype-conservation caused by EPO in human mesenchymal dermal stem cells grown under hypoxic conditions and in IL-6 enriched media [4].

Additionally, there are numerous publications reporting EPO’s function as a protective molecule of capillary endothelial cells, which under hypoxic stress would normally become apoptotic, thus improving the blood supply in the surrounding tissues [5]. In many tissues, inhibition of apoptosis is induced directly via a phosphorylation cascade initiated by the EPO receptor. Numerous pathways can be attributed to phosphorylation of janus kinase 2 (JAK-2) and subsequent phosphorylation of signal transducers and activators of transcription (STATs) and B-cell lymphoma extra-large (BCL-XL) or are mediated via phosphatidylinositol-3 kinase (protein kinase A) (PI3K-Akt) and mitogen-activated protein kinase (MAPK) pathways [6,7]. STAT then evokes apoptotic inhibition in the nucleus. Furthermore, Akt (protein kinase B) is activated, which plays a central role in cell survival [8].

Several authors report that EPO also triggers a variety of growth factors (see Table 1). Since we are focusing our discussion onto factors which are known to be involved in wound healing, the present work describes a selected number of growth factors and their effects.

**Table 1:** Most important effects of EPO on different growth factors in wound healing

**Factor**	**Action**	**EPO action**	**Tissue, organ, species**	**Reference**
TNF-α	Primary-injury response	Antagonist	Neuronal tissue, brain, mice	[9]
IL-2, -6, -8	Proinflammatory cytokines	Inhibition	Human histiocytic lymphoma cells	[10]
				[11]
γ-interferon	Proinflammatory cytokine	Inhibition	Human histiocytic lymphoma cells	[10]
MIP 1 u. 2	Proinflammatory protein	Inhibition	Cartilage, mice (arthritis model)	[12]
Myeloperoxidase	Invasion-marker for macrophages	Inhibition	Neuronal tissue, brain, mice	[13]
IL-1β	Proinflammatory cytokine	Inhibition	Human histiocytic lymphoma cells	[10]
γ-interferon	Proinflammatory cytokine	Inhibition	Human leucocytes	[14]
VEGF	Angiogenesis	Stimulation	Skin, microvessels, mice	[15]
eNOS	Recruitment of EPCs	Stimulation	Skin, microvessels, mice	[16]
EGF	Angiogenesis	Stimulation	Heart, microvessels, neovasculature, rat	[17]
HIF-1α / HIF-2α	Adaption to hypoxia, stimulation of EPO and VEGF	Stimulation	Human hepatoma cell lines	[18]
EPAS 1	Adaption to hypoxia	Stimulation	Cortical astrocytes	[19]
			Cortical astrocytes	[19]
iNOS	Relaxation of smooth muscle cells	Stimulation	Skin, microvessels, mice	[15]
BCL-Xl	Anti-apoptotic	Stimulation	Erythroid progenitor cell line, mice	[20]
IGF-I	Anti-apoptotic	Stimulation	Astrocytoma cell line	[21]
	cell proliferation			
Caspase-3	Apoptosis	Inhibition	Kidney, rat	[22]
BCL-2	Anti-apoptotic	Stimulation	Erythroid progenitor cell line, mice	[23]
	proliferation			
hsp 70	Intracellular protective processes	Stimulation	Human TF-1 cell line	[24]

BCL: B-cell lymphoma; EGF: endothelial growth factor; eNOS: endothelial nitric oxide synthase; EPAS: endothelial PAS domain-containing protein; EPC: endothelial progenitor cell; hsp: heat shock protein; IGF: insulin-like growth factor; iNOS: inducible nitric oxide synthase; MIP: macrophage inflammatory protein; TNF: tumor necrosis factor; VEGF: vascular endothelial growth factor.

Several publications indicate that the actions of specialized proinflammatory cells, such as leukocytes and macrophages, are down-regulated by EPO: the production of IL-2, IL-6, IL-8, γ-interferon and tumor necrosis factor α (TNF-α) is antagonized [10,14,25].

Normal human skin contains EPO receptors and is able to synthesize EPO. In culture, under hypoxic conditions, EPO synthesis increases [26].

In a mouse model the combined presence of EPO receptor (EPOR) and the EPO-hetero-receptor could be demonstrated in healthy and scalded skin. Additionally, after systemic EPO application the EPOR was significantly down-regulated in the healthy skin; by contrast, in the scalded skin, the expression rate stayed unchanged [27].

Several investigations on the effects of EPO on wound healing have shown predominantly elevated vascular endothelial growth factor (VEGF) and endothelial growth factor levels (and resulting elevated capillary density) in treatment groups compared to controls. This was emphasized by increased cluster of differentiation 31 (CD31) gene expression. Higher protein and collagen densities, as well as the greater mechanical stability of fresh cicatrices, were found in treatment groups as well [28].

One of the first research projects to examine the effects of EPO on wound healing, used fibrin-filled subcutaneously implanted chambers. These chambers were then injected with an EPO/fibrin mixture with different concentrations of EPO. The formation of granulation tissue was stimulated by the administration of EPO. When an anti-EPO or anti-EPO receptor antibody was added, the formation of granulation tissue deteriorated [29].

An *in vitro* study examined the effects of EPO and EPO receptors in human hair follicles. It was shown that hair follicle cells produce EPO and EPO receptors and that EPO shows its typical tissue-protective effects. Protective effects against chemotherapy-induced apoptosis of hair follicle cells was demonstrated [26]. Skin-derived EPO appears to act locally, while it is less important for the systemic EPO response to acute hypoxia in healthy humans [30].

The effects of subcutaneous EPO on dermal regeneration were also investigated using nude mice. In this model, the healing of a full thickness defect was traced directly into the skin-fold chamber in nude mice. It was shown that with EPO the healing process not only improved but was also accelerated depending on the dosage [31].

In another mouse model, after thermal trauma and subsequent EPO treatment, improved and accelerated wound healing was observed compared to the control group [32]. This was marked by increased epithelial proliferation, maturation of the extra-cellular matrix, increased angiogenesis and increased capillary density, identified by high CD31, VEGF values and eNOS activity. When mice were administered anti-EPO antibodies, wound healing deteriorated below that of the control group. After thermal trauma in mice and subsequent EPO treatment, mice exhibited improved and accelerated re-epithelialization as well as faster wound healing compared to control groups. This was characterized by increased epithelial proliferation, and maturation of extra-cellular matrices, as well as increased angiogenesis and capillary density detectable via high CD31 and VEGF values and eNOS activity [32].

The above findings lead to the assumption that EPO represents a new, effective therapeutic opportunity for the treatment of acute dermal thermal injuries.

## Methods/Design

This study is conducted according to globally accepted standards of good clinical practice (as defined in the ICH E6 Guideline for Good Clinical Practice, 1 May 1996), in agreement with the Declaration of Helsinki and in keeping with local regulations. It has full approval of the designated ethic committee (at the University of Lübeck, Ratzeburger Allee 160, Building 21, 23562 Lübeck, Germany) and is funded by the German Federal Ministry of Education and Research. Additionally, the study received support for medication costs from Heraeus Holding GmbH.

### Objectives

The study objectives are to demonstrate a cytoprotective and regenerative effect of erythropoietin in thermally injured patients in terms of reduced morbidity and mortality and to better understand the cellular mechanisms of erythropoietin in skin graft donor sites (SGDSs) and second-degree wounds (SDWs).

### Study design

This is a prospective, randomized, double-blind, multi-center clinical trial, conducted in 13 burn care centers in Germany. In total, 150 adult patients with burn and scald injuries will be included.

Via a centralized randomization list, patients are randomly allocated to the drug- or the placebo-treated group. Patients receive normal state-of-the-art burn care treatment plus study medication every other day. Study wound sites (SGDCs and SDWs) are treated with standard wound dressings. Dressing changes, wound documentation, photo documentation, biopsies, control of safety parameters, study medication and other relevant actions are strictly regulated in the study protocol. The acute treatment and observational phase is 28 days, with follow-up controls at Day 42, after 6 months and 12 months after inclusion.

### Patient population

Adult patients between 18 and 75 years of age with deep burns (deep second (2b) or third degree) or scalding injuries between 5% and 60% of total body surface (TBS) (patients under 40 can have more than a 60% TBS injury), and where a necrectomy and consecutive split skin graft transplantation is indicated, are to be included in the trial. Patients who are either pregnant or breastfeeding are to be excluded. Informed consent in written form must be given before the first study-specific action, by the patient, the patient’s legal representative or via the *Heidelberger Verfahren*. The *Heidelberger Verfahren*, first described by Brückner et al. [33], allows recruitment of patients who are unable to give informed consent themselves and where there is insufficient time to appoint a legal representative. In such cases, a judge, in consensus with the ethics committee and the judicial court, decides whether a patient can take part in the trial and gives the patient’s next-of-kin the authority to give informed consent on behalf of the patient. However, in cases where a next-of-kin is not available or unknown, the judge has the legal authority to give informed consent in advance and simultaneously a legal representative will be designated, who will subsequently give informed consent. Additionally as soon as the patient is able to give informed consent, he or she will be asked to do so.

### Inclusion criteria

3° burn and scalding thermal injuries obligate, 2° burn and scalding thermal injuries optional, which require operations including split skin harvesting and grafting

Men and women, age ≥ 18 and ≤ 75 years

Secure contraception.

### Exclusion criteria

Admission later than 24 hours after injury.

Hematological disorders (anemia, lymphoma, leukemia, inborn coagulation diseases).

Pregnancy or breast-feeding.

Estimated survival shorter than one week (abbreviated burn severity index (ABSI) > 12) in patients older than 40 years of age.

Body weight < 50 kg or > 110 kgTotal burn surface area involved less than 60% in patients older than 40 years of age.

In patients below 40 years of age no limitation of maximum burned body surface and no limitation of ABSI score will be considered.

Upper lateral thighs of both legs thermally injured.

Subject is the investigator or any sub-investigator, research assistant, pharmacist, study coordinator, other staff or relative thereof directly involved in the conduct of the protocol.

Subject is unlikely to comply with protocol, e.g., uncooperative attitude, inability to return for follow-up visits, and unlikelihood of completing the study.

Treatment with any investigational product in the last 12 months before study entry.

Treatment with any immunosuppressive therapy, cancer-related chemotherapy or radiation therapy in the past 12 months.

History of hypersensitivity to the investigational products.

Likelihood of requiring treatment during the study period with drugs not permitted by the clinical study protocol.

Clinically relevant cardiovascular (s.p. cardiac infarction, coronary heart disease (CHD), treatmenresistant hypertension, thromboembolic disease, thromboembolic events shortly before admission), hepatic (Child B or C liver disease), endocrine (morbid obesity (BMI > 40)) or systemic (cancer) disease (malignoma).

Epileptiform diseases.

Phenylketonuria.

HIV disease, AIDS.

Informed consent missing.

### Treatment

Randomized patients receive state-of-the-art burn care treatment including (depending on the clinical situation of the individual patient) analgosedation, respiration, escharectomy and split skin grafting or, if necessary, temporary wound closure. General wound care and wound dressings can be used, as they are standard practice in the study centers. The only exceptions are the studied split SGDS, the studied second-degree wound and the studied third-degree wound, which have to be treated as described in detail in the study protocol.

The study medication is either 150 IU recombinant human EPO (rhEPO) (NeoRecormon Multidose 50,000 IU, Roche, Grenzach-Wylen, Germany) per kilogram of body weight or a matched placebo (see Table 2), which is administered every other day subcutaneously for 21 days. The split SGDS is to be generated on Day 2 of study treatment and special wound dressings with a polyurethane foil have to be applied according to the trial protocol. Dressing changes and wound controls are made at specified time points. Patients receive a standardized anticoagulant therapy with body weight-adjusted intravenous heparin.

**Table 2:** Composition of placebo solution

**Substance**	**Amount**
NaCl	8.24 g
Benzyl alcohol	3.67 g
Benzalkonium chloride	0.58 g
H_2_O ad injectionem	Ad 1,000 ml

### Measurements

Race, gender, age, height, weight, TBS burned percentage, severity of burn (grades 1, 2a, 2b or 3), ABSI and concomitant illnesses or injuries will be documented upon admission. Woman of reproductive age will undergo a pregnancy test to check for an unknown pregnancy.

Daily controls of the organ failure parameters, using the sequential organ failure assessment (SOFA), and safety relevant parameters will be performed. The wound will be clinically assessed and photographs taken according to the protocol. Special EPO-related blood samples will be taken once a week for four consecutive weeks. Punch biopsies will be performed from the SGDS and the SDW four times and once from healthy skin as a control.

### Primary endpoint

The primary endpoint is the time until complete re-epithelialization of the SGDS at a specified location on the lateral upper thigh.

### Secondary endpoints

Time until complete wound healing of type 2a SDW

Time until complete wound healing of skin graft.

Cellular and molecular regenerative effects in SGDS and type 2a SDW, Endothelial.

Progenitor Cell (EPC) recruitment, EPO receptor up-or down-regulation, protein-, RNA- and microRNA- expression patterns

Quality of scar formation in conservatively and operatively treated wound locations.

Number of packed red cells units, which are transfused during the treatment interval- Organ dysfunction parameters- Quality of life 12 months after trauma (SF-36)- Mortality (within 21 days, during the hospital stay and 1 year on).

Adverse events (AEs), Serious Adverse Events (SAEs), Suspected Unexpected Serious.

Adverse Reactions (SUSARs)

Gender differences in monitored data.

### Safety data

The safety endpoints include AEs, SAEs, SUSARs, laboratory test results and vital signs. The data safety monitoring board (DSMB) will supervise the occurrence of AEs, SAEs, SUSARs and overall safety parameters.

A group of independent experts will form the Safety Monitoring Board. The DSMB will independently review the safety data for the duration of the study. In addition, the coordinating investigator will inform the DSMB of any SUSARs with a written report.

The DSMB will give advice when specific actions are necessary. The study sites will be immediately informed of the procedures. The DSMB will discontinue the study if a quantitative accumulation of SAEs is observed. In addition, if there is an unexpected accumulation of SAEs other than mentioned above during the study, the DSMB will discontinue the study. Additionally, annual safety and progress reports have to be prepared for the authorities and the independent ethics committees.

### Statistical considerations

#### Sample size justification

Under conventional treatment, complete re-epithelialization of split skin graft donor sites occurs after a mean of 10 to 15 days with a standard deviation (SD) of about 6 days, depending on the donor site and the patient’s general condition. We expect that EPO treatment can reduce the total healing time by at least 4 days. Sample size calculation with alpha = 0.05 and beta = 0.10 thus indicates that 48 evaluable patients per group are necessary. In order to have 96 patients with valid data for the intention-to-treat analysis of the primary outcome, we aim to include 150 patients. This allows for 54 dropouts (34%).

#### Statistical analysis

Analyses will include all randomized patients with at least one exposure to the study drug. In addition to the intention-to-treat population, an exploratory analysis of all patients completing the study as planned (per protocol analysis) will be carried out. Standard statistical testing will be used to examine the superiority of one treatment arm. Testing will be adjusted for two stratification factors: ABSI group and hospital. The primary endpoint for the time taken for re-epithelialization of the skin graft donor site will be analyzed as a continuous variable with normal distribution. A safety analysis will be carried out for all adverse events (regardless of severity). According to current standards, the safety analysis will exclude those patients who failed to receive any study drug dose. A list of all adverse events will be provided and periodically distributed to the DSMB. The two-sided significance level for the final analysis is 0.05.

## Discussion

The primary aim of this study is to evaluate the effect of repetitive low dose rhEPO administered within 21 days in severely thermally injured patients. There are several reasons to believe that repetitive low dose rhEPO will improve wound healing in this patient population without severe adverse events or other grave complications. rhEPO is a well-known drug and has been used for nearly 30 years in daily clinical practice to treat anemia [34]. Thus the risk/benefit ratio for patients is relatively concrete to estimate, and will be discussed later. In addition the erythropoietic response to EPO is markedly decreased in thermally injured patients [35,36].

During the last two decades of the 20th century, scientific interest has turned towards treating burned patients with EPO, especially those patients who refuse blood transfusions and blood products due to religious reasons [35,36]. Several single-case reports have been published and two trials have been performed on thermally injured patients in intensive burn care units in an attempt to improve erythropoiesis. Both trials failed to show that doses between 300 and 500 IU rhEPO per kilogram of body weight daily for 7 days could significantly reduce the amount of required red blood cell transfusions. No statistically significant differences in hemoglobin, hematocrit, reticulocyte count, ferritin, serum iron, total iron binding capacity or transfusion requirements were found in either study. The effects on wound healing were not within the scope of the studies and therefore not monitored. In patients with burns involving 25% to 35% of total body surface, the reticulocyte counts were statistically significantly higher. In one trial it became evident that iron supplementation needs to be performed in patients receiving 500 IU EPO/kg body weight daily for 7 days to avoid iron depletion. In the other trial, three patients in the rhEPO group suffered venous thrombosis, compared with one patient in the placebo group. No further adverse side effects, possibly related to EPO, were encountered in either trial.

In 2005, an *in vitro* study showed that the serum of burned mice had elevated EPO levels, and bone marrow cell cultures treated with this serum reacted with increased proliferation rates [37].

The fact that no increase in hemoglobin, hematocrit, reticulocyte or erythrocyte count was found in either study is an especially interesting aspect regarding the treatment of burned patients; besides the well-known fact of decreased EPO efficacy regarding erytropoiesis in inflammation and in trauma situations of the organism, no explanation for this phenomenon has been given so far.

Known complications of EPO treatment are thromboembolic events. A large multi-center trial investigating the safety of EPO administration to poly-traumatized patients demonstrated EPO is safe and beneficial for these patients and that thromboembolic events were reduced to ranges seen in poly-traumatized patients not receiving EPO when combined with an appropriate anti-thrombotic therapy [38]. As the thromboembolic risk of thermally injured patients is comparable to that of poly-traumatized patients, we conclude that EPO administration in thermally injured patients is safe when combined with an adequate anti-thrombotic prophylaxis as it is done in our trial.

There are two further well-known adverse events related to EPO. The first is the increase in blood pressure in already hypertonic patients [39] with the consequence that therapy resistant arterial hypertension is an exclusion criterion of the trial. The second is the unclear situation of EPO in patients with malignant tumors. In several trials it was shown that the mortality rate of EPO-treated groups is higher than for untreated controls [39]. Therefore, a known malignancy is also an exclusion criterion of the trial.

Age, nutritional status, affected total body surface, gender, concomitant illnesses, inhalation injury or other concomitant trauma, as well as wound infections, have a large influence on the patient’s prognosis, survival rate and wound healing. Therefore we considered all of these factors carefully and discussed their relevance for our primary and secondary endpoints. As we are unable to foresee which patient group will profit most, we consider an exclusion of one of this groups as unethical.

Dermal thermal traumas are commonly seen in two age groups of adults. The first group comprises young adults aged between 18 and 28 and the second group comprises elderly persons aged over 65 years. As we are not able to foresee which of these groups would benefit most from pro-regenerative treatment, we decided to include patients aged 18 to 75 in our study [40].

The nutrition of burns patients is also a very extensively discussed topic, but so far no official guidelines exist and scientific evidence is not satisfactory. Thus we accepted the regulations of the burns centers and their in-house nutrition regimes. The authors expect that this will influence the results and they will consider this very carefully during the evaluation. This is one of the reasons why we have an adjustment for the centers (hospital).

The severity of the injury (affected total body surface and burn degree) has a significant influence on the prognosis of the patient, especially in combination with age and gender. Here again we are unable to foresee which patient group will profit most.

Concomitant illnesses are important factors especially if they interact with EPO. Thus, as stated above, we exclude patients with illnesses such as hypertension, malignant diseases, etc.

Interesting first reports concerning the prevention of burn progression by systemic administration of EPO in a rat model have been given as congress reports. These promising results need further investigation [41].

We hypothesize, therefore, that EPO can protect the epidermis, dermis and its vital structures – especially the capillaries and blood vessels – from further damage, for example, further burn progression. This very often increases the depth of the thermal injury and, therefore, accelerates the problem. EPO has to be administered in a time frame during which its anti-apoptotic action can prevent the increase of the penumbra.

Overall, we expect EPO treatment in thermally injured patients to be safe and beneficial when combined with an adequate anti-thrombotic prophylaxis, as has already been seen for decades in patients with anemia due to renal failure.

In conclusion, we expect that because of the tissue and cell-protective effects (anti-apoptotic, anti-inflammatory, stem cell recruiting and pro-angiogenic) of EPO, its biomimetic effects will be useful in the treatment of acute thermal dermal injuries.

## Trial status

This is an investigator-initiated Phase 2a trial funded by the German Federal Ministry of Education and Research. Full approval has been given by the ethics committees. The trial is in the recruitment phase.

## Abbreviations

ABSI: abbreviated burn severity index; AE: adverse event; BCL: B-cell lymphoma; BCL-XL: B-cell lymphoma extra-large; CD: cluster of differentiation; DSMB: data safety monitoring board; EGF: endothelial growth factor; eNOS: endothelial nitric oxide synthase; EPAS: endothelial PAS domain-containing protein; EPC: endothelial progenitor cell; EPO: erythropoietin; EPOR: EPO receptor; Hsp: heat shock protein; IGF: insulin-like growth factor; IL: interleukin; iNOS: inducible nitric oxide synthase; IU: international units; JAK: janus kinase; MAPK: mitogen-activated protein kinase; MIP: macrophage inflammatory protein; PI3K-Akt: phosphatidylinositol-3 kinase (protein kinase A); rhEPO: recombinant human EPO; SAE: serious adverse event; SDW: second-degree wound; SGDS: skin graft donor site; SOFA: sequential organ failure assessment; STAT: signal transducer and activator of transcription; SUSAR: suspected unexpected serious adverse reaction; TBS: total body surface; TNF: tumor necrosis factor; VEGF: vascular endothelial growth factor.

## Competing interests

The authors declare they have no conflicting interests.

## Authors’ contributions

CG was the vice-coordinating investigator for the trial and the main author of the article. AB was responsible for the tissue analyses of the trial, and made a substantive intellectual contribution, having developed the hypothesis together with H-GM and WJ, and performed all pre-clinical experiments. UD made a substantial intellectual contribution during the preparation phase of the trial protocol. CO was responsible for the data management of the trial and was the author of the data management section. SE was responsible for organizational and formal trial management and was the co-author of the study description. VK was responsible for the statistics and the biometry of the trial and was the author of the statistics section. WJ was responsible for the EPO analyses of the trial and made a substantive intellectual contribution, having developed the hypothesis together with AB and H-GM. H-GM was the coordinating investigator for the trial and made a substantive intellectual contribution, having developed the hypothesis together with AB and WJ, and performed all pre-clinical experiments and clinical case studies ahead of the trial. All authors read and approved the final manuscript.
